# Supplementary material for: Prevalence and factors associated with illicit substance use among persons with Schizophrenia at a Tertiary Referral Hospital in Zambia
Source: PLOS Glob Public Health. 2026 Jan 12;6(1):e0005024. doi: 10.1371/journal.pgph.0005024 (PMC12795356; doi:10.1371/journal.pgph.0005024)
Supplement: S1 Table — (DOCX) [file pgph.0005024.s002.docx]

**Supplementary Table 1**

| Variable | VIF |
| --- | --- |
| age_years | 15.923495088940964 |
| residence | 15.42878739138858 |
| disorganized_behaviour | 11.717979258774818 |
| sex | 10.055930782941289 |
| education | 9.227281157850777 |
| hallucinations | 5.805923277501574 |
| number_of_hospitalizations | 5.072462796958644 |
| patient_adherent_to_medica | 3.6023245091732763 |
| is_patient_aware_of_their | 3.1867900990475984 |
| married | 2.922568912263822 |
| delusions | 2.6671924223984593 |
| cognitive_impairement | 2.6579582705887694 |
| disorganized_speech | 2.6573693905471965 |
| violent_behavior | 2.446430544612004 |
| both_parents_alive | 2.2250921677152005 |
| alcohol_use | 2.1913062022949266 |
| both_parents_deceased | 2.0986398741159564 |
| smoking | 2.046329291095575 |
| employed | 2.030195617569146 |
| has_the_patient_consulted | 1.767787224396799 |
| negative_symptoms | 1.5046590031870548 |
| family_history_of_other_ps | 1.4320368855343393 |
| history_of_self_harm | 1.2458240342069182 |
| suicidal | 1.1434647557447366 |
| family_history_of_schizoph | 1.0990720964661578 |
