# Supplementary material for: Prevalence and factors associated with illicit substance use among persons with Schizophrenia at a Tertiary Referral Hospital in Zambia
Source: PLOS Glob Public Health. 2026 Jan 12;6(1):e0005024. doi: 10.1371/journal.pgph.0005024 (PMC12795356; doi:10.1371/journal.pgph.0005024)
Supplement: S2 Table — (DOCX) [file pgph.0005024.s003.docx]

**Supplementary Table 2**

Logistic Regression Model Results:


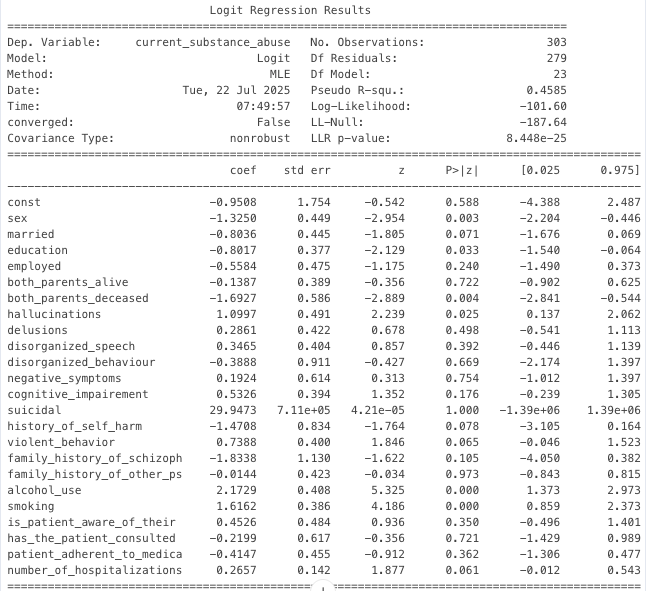


**Hosmer-Lemeshow Goodness-of-Fit Test**

Chi-square statistic: 8.5747; Degrees of freedom: 8; P-value: 0.3794

Result: Good fit (p > 0.05, fail to reject null hypothesis)

Significant Predictors (p < 0.05):


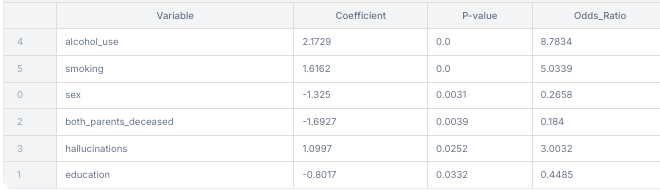


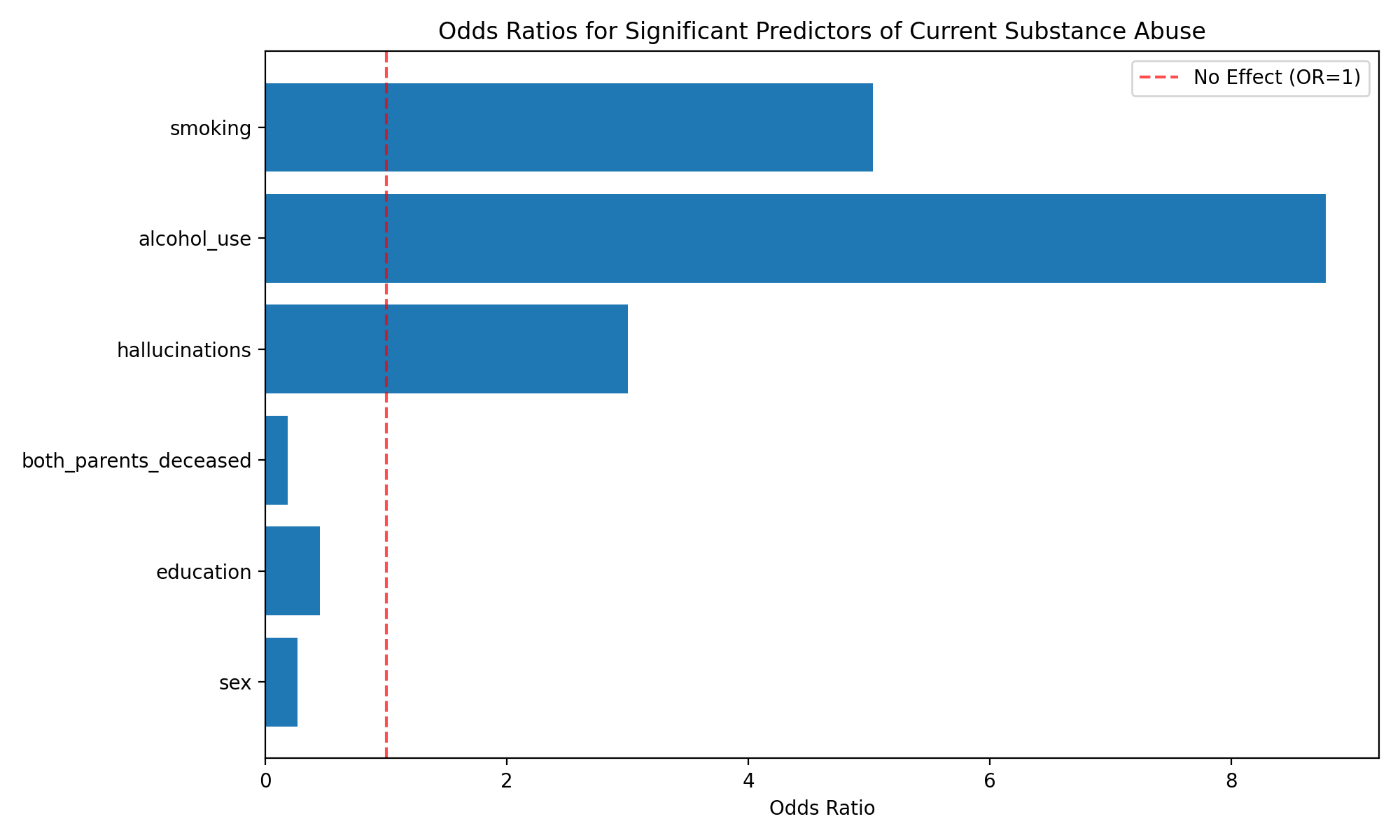


## Key Findings:

**Model Fit**: The Hosmer-Lemeshow test indicates good model fit (p = 0.3794 > 0.05), meaning we fail to reject the null hypothesis of adequate fit.

**Significant Predictors** (p < 0.05):

- **Alcohol use**: Strongest predictor (OR = 8.78) - patients with alcohol use are nearly 9 times more likely to have current substance abuse
- **Smoking**: Second strongest (OR = 5.03) - smokers are 5 times more likely to have substance abuse
- **Hallucinations**: Increases odds by 3 times (OR = 3.00)
- **Sex**: Males have lower odds (OR = 0.27) - being female increases substance abuse risk
- **Both parents deceased**: Protective factor (OR = 0.18) - surprisingly reduces substance abuse risk
- **Education**: Higher education is protective (OR = 0.45)

**Model Performance**: The model shows good discriminative ability with 83% accuracy, pseudo R² of 0.46, and reasonable precision/recall for both classes.
