# Supplementary material for: Prevalence and factors associated with illicit substance use among persons with Schizophrenia at a Tertiary Referral Hospital in Zambia
Source: PLOS Glob Public Health. 2026 Jan 12;6(1):e0005024. doi: 10.1371/journal.pgph.0005024 (PMC12795356; doi:10.1371/journal.pgph.0005024)
Supplement: S3 Table — (DOCX) [file pgph.0005024.s004.docx]

**Supplementary Table 3**

MODEL PERFORMANCE COMPARISON:

| Model | Variables | Pseudo_R2 | AIC | Log_Likelihood |
| --- | --- | --- | --- | --- |
| Full Model | 23 | 0.4585 | 251.201 | -101.6005 |
| Significant Only | 6 | 0.3599 | 254.2366 | -120.1183 |
| Forward Selection | 7 | 0.3846 | 246.9519 | -115.4759 |
| Backward Elimination | 7 | 0.3863 | 246.3311 | -115.1655 |
| Clinical Significance | 7 | 0.345 | 261.7988 | -122.8994 |

Best model based on AIC:

Backward Elimination

CROSS-VALIDATION COMPARISON

Cross-Validation Results (5-fold):

| Mean_CV_Accuracy | Std_CV_Accuracy |
| --- | --- |
| 0.7627 | 0.0504 |
| 0.7757 | 0.0521 |
| 0.7593 | 0.0357 |
| 0.7726 | 0.0572 |

DETAILED ANALYSIS OF BEST MODEL (BACKWARD ELIMINATION)

| Variable | Coefficient | P_value | Odds_Ratio | 95% CI_Lower | 95% CI_Upper |
| --- | --- | --- | --- | --- | --- |
| alcohol_use | 1.8058 | 0.0 | 6.085 | 3.1428 | 11.7817 |
| smoking | 1.5696 | 0.0 | 4.8045 | 2.4403 | 9.4593 |
| sex | -1.298 | 0.0014 | 0.2731 | 0.1232 | 0.6051 |
| married | -0.9241 | 0.0086 | 0.3969 | 0.1993 | 0.7904 |
| education | -0.8032 | 0.009 | 0.4479 | 0.2452 | 0.8183 |
| both_parents_deceased | -1.2256 | 0.0111 | 0.2936 | 0.114 | 0.7558 |
| number_of_hospitalizations | 0.2576 | 0.0384 | 1.2939 | 1.0139 | 1.6511 |

GOODNESS OF FIT TEST (BACKWARD ELIMINATION MODEL)

Hosmer-Lemeshow Test Results:

Chi-square statistic: 18.2212

Degrees of freedom: 8

P-value: 0.0196

Interpretation: Poor fit (p <= 0.05)

=== FINAL MODEL RECOMMENDATIONS ===

BEST MODEL: Backward Elimination Model

- Variables: 7
- Pseudo R-squared: 0.3863
- AIC: 246.33 (lowest among all models)
- Cross-validation accuracy: 75.93%

KEY FINDINGS FROM BEST MODEL:

1. STRONGEST PREDICTORS (highest odds ratios):
   - Alcohol use: OR = 6.09 (95% CI: 3.14-11.78)
   - Smoking: OR = 4.80 (95% CI: 2.44-9.46)
2. PROTECTIVE FACTORS (OR < 1):
   - Being male: OR = 0.27 (95% CI: 0.12-0.61)
   - Being married: OR = 0.40 (95% CI: 0.20-0.79)
   - Higher education: OR = 0.45 (95% CI: 0.25-0.82)
   - Both parents deceased: OR = 0.33 (95% CI: 0.11-0.76)
3. RISK FACTORS:
   - Number of hospitalizations: OR = 1.29 (95% CI: 1.01-1.65)

MODEL LIMITATIONS:

- Hosmer-Lemeshow test suggests poor calibration (p = 0.0196)
- May need additional variables or interaction terms
- Consider non-linear relationships

The analysis shows that alcohol use and smoking are the strongest predictors of current substance abuse, with odds ratios of 6.09 and 4.80 respectively. Interestingly, several factors appear protective, including being male, married, having higher education, and having both parents deceased. The backward elimination model performed best overall with the lowest AIC, though the Hosmer-Lemeshow test suggests some calibration issues that might be addressed with additional model refinements.
