# Supplementary material for: Prevalence and factors associated with illicit substance use among persons with Schizophrenia at a Tertiary Referral Hospital in Zambia
Source: PLOS Glob Public Health. 2026 Jan 12;6(1):e0005024. doi: 10.1371/journal.pgph.0005024 (PMC12795356; doi:10.1371/journal.pgph.0005024)
Supplement: Attachment — Submitted filename: Comment by Tamene Berhanu May 29, 2025.docx [file pgph.0005024.s006.docx]

**May 29, 2025**

**PLOS Global Public Health**

Prevalence and Factors Associated with Substance Abuse among Schizophrenic Patients at LUTH in Zambia

**--Manuscript Draft--**

**Manuscript Number: PGPH-D-25-01149**

**Reviewed by Tamene Berhanu Alaho (**Mental Health Specialist**)**

**Overall:** I would like to thank for inviting to review this manuscript. This study will have great value in sub-Saharan countries to enhance awareness and prevention strategies for mental health wellbeing and its association with substance use disorders. I hope this manuscript described well.

I have few comments, find it below

**Comments**

**Abstract section**

1. The word alcohol (kachasu) mentioned, what does ‘kachasu’ mean? Is it Zambian language for alcohol or not? If you misspelled kaht use revise it
2. DSM-5 or DSM-5TR currently defines substance use rather than substance abuse. Since the word abuse is inappropriate term and increase negative view on substance users. It is better to replace substance abuse by substance use in your whole document
3. ‘Ethical approval and informed consent were obtained’ put this statement in ethical consideration section only.

**Introduction/Background**

1. ‘Yet the country has only five psychiatrists’. This statement is for whole Zambian country or the only for University Teaching Hospital (LUTH). Make clear this statement otherwise it seems like exaggeration of the problems

**Method**

1. The data collected from patient medical recorded or secondary data might be prone to biased information, what you did to minimize bias in your study? Describe this carefully in the limitation section
2. Describe smartly about the data quality assurance since the secondary data need more attention to minimize missed information.
3. What you did for participants who had substance use disorder after screening? If you did something, please specify it?

**Result section**

1. For the variables Age and Number of hospitalizations has reference category not specified why? Use the same format for all variables.

**Discussion**

1. ‘In this study the prevalence of substance abuse was 31.1% (94/303). Alcohol use (60.2% vs.46 13.7% non-users, p<0.001) and smoking (61.5% vs. 15.1% non-smokers, p<0.001)’. Please discuss this finding with others study which have similar, higher or below finding compared to this study by giving convincing scientific evidences

**Limitations, Recommendation and conclusion**

1. Describe limitation in detail
2. Conclusion Should be smart, short and prices
3. Recommendation based on finding, be carefully about this
4. Is it ASSIT valid in Zambia? If not valid it is better to recommend future study to do validation.

**Overall:** be carefully about grammar, paraphrasing and correct reference

**Finally**; - thank you for your contribution to existing body of knowledge’s and submitting your manuscript to PLOS Global Public Health
