# Supplementary material for: Prevalence and factors associated with illicit substance use among persons with Schizophrenia at a Tertiary Referral Hospital in Zambia
Source: PLOS Glob Public Health. 2026 Jan 12;6(1):e0005024. doi: 10.1371/journal.pgph.0005024 (PMC12795356; doi:10.1371/journal.pgph.0005024)
Supplement: Attachment — Submitted filename: Response to Reviewers.docx [file pgph.0005024.s007.docx]

**RESPONSES TO EDITOR AND REVIEWER COMMENTS**

We sincerely thank the reviewers for their thorough assessment and constructive feedback, which have significantly strengthened our manuscript. We have carefully addressed all comments and provide a point-by-point response below. Revised sections are tracked in the manuscript file.

When submitting your revision, we need you to address these additional requirements.

1. Please insert an Ethics Statement at the beginning of your Methods section, under a subheading 'Ethics Statement'.

**Response from authors:** we have now moved the ethics statement to the beginning of the methods section.

2. We note that your Data Availability Statement is currently as follows: All relevant data are within the manuscript and its supporting information files.

Please confirm at this time whether or not your submission contains all raw data required to replicate the results of your study. Authors must share the “minimal data set” for their submission. PLOS defines the minimal data set to consist of the data required to replicate all study findings reported in the article, as well as related metadata and methods (https://journals.plos.org/plosone/s/data-availability#loc-minimal-data-set-definition).

**Response from authors:** Thank you, we have since attached the raw data to this submission as S2 Data.

**Review Comments to the Author**

Reviewer 1
1. The word alcohol (kachasu) mentioned, what does ‘kachasu’ mean? Is it Zambian language for alcohol or not? If you misspelled kaht use revise it.

**Response from authors:** We appreciate this query. "Kachasu" refers to a traditionally fermented, highly intoxicating distilled alcoholic spirit commonly consumed in Zambia. We have clarified its definition in the Introduction (Lines 55–56) to ensure readers understand this culturally specific term.

2. DSM-5 or DSM-5TR currently defines substance use rather than substance abuse. Since the word abuse is inappropriate term and increase negative view on substance users. It is better to replace substance abuse by substance use in your whole document

**Response from authors:** Thank you for highlighting this important distinction. We have replaced all instances of "substance abuse" with "substance use" in the manuscript to align with current diagnostic terminology and reduce stigma.

3. ‘Ethical approval and informed consent were obtained’ put this statement in ethical consideration section only.

**Response from authors:** We have relocated this statement to the "Ethics Statement" subsection (Methods) and removed it from the Abstract to maintain appropriate section alignment.

4. ‘Yet the country has only five psychiatrists’. This statement is for whole Zambian country or the only for University Teaching Hospital (LUTH). Make clear this statement otherwise it seems like exaggeration of the problems

**Response from authors:** Thank you so much. We have revised the statement, which was based on the 2017 statistics, and included the latest statements. However, the number of psychiatrists remains very low with the latest statistics indicating a rise to 10 in the country.

5. The data collected from patient medical recorded or secondary data might be prone to biased information, what you did to minimize bias in your study? Describe this carefully in the limitation section.

**Response from authors:** We acknowledge this concern. The Limitations section now explicitly states: "Recall and documentation biases may have led to underreporting, particularly given stigma and clinician workload constraints at LUTH" (Lines 531–534). We further note that exclusion of incomplete records may introduce selection bias.

6. Describe smartly about the data quality assurance since the secondary data need more attention to minimize missed information.

**Response from authors:** We have expanded the Methodology to detail quality controls: "Data quality was ensured through predefined abstraction criteria, dual independent review of 10% of records, REDCap audit trails, and exclusion of records missing >20% of core variables" (Lines 177–180).

7. What you did for participants who had substance use disorder after screening? If you did something, please specify it?

**Response from authors:** We clarify that as a retrospective study, no interventions were administered by researchers. However, we note: "Per LUTH protocol, patients identified with substance use receive brief interventions and referrals to addiction services" (Lines 192–193).

8. For the variables Age and Number of hospitalizations has reference category not specified why? Use the same format for all variables.

**Response from authors:** Continuous variables were analyzed per unit increase (e.g., per additional hospitalization) and thus do not require reference categories. We have standardized the presentation of results in Table 2 to avoid confusion.

9. In this study the prevalence of substance abuse was 31.1% (94/303). Alcohol use (60.2% vs.46 13.7% non-users, p<0.001) and smoking (61.5% vs. 15.1% non-smokers, p<0.001)’. Please discuss this finding with others study which have similar, higher or below finding compared to this study by giving convincing scientific evidences.

**Response from authors:** Thank you so much. We have revised our discussion and included research findings from Nigeria, Ethiopia as well as Sub Saharan Africa that were different in comparison to our findings. We now contextualize our findings: "The prevalence is lower than SSA averages (58–65%) but aligns with Nigerian (43%) and Ethiopian (46.8%) studies—potentially reflecting Zambia’s strict criminalization or underdetection in records" (Lines 396–399).

10. Describe limitation in detail

**Response from authors:** Thank you so much. We have revised and included more details to the limitations. The Limitations section now includes: "Unmeasured confounders (e.g., income, trauma history), ASSIST’s unvalidated status in Zambia, and limited generalizability beyond tertiary settings" (Lines 535–540).

11. Conclusion should be smart, short and prices

**Response from authors:** Thank you for the comment. We have revised the conclusion to ensure that it is smart, short and precise and also in line with our findings. The Conclusion has been condensed to: "Male sex, alcohol, and smoking are key risk factors; female sex, marriage, parental loss, and education confer protection. Integrated gender-specific interventions, socioeconomic support, and addiction services for high-hospitalization patients are warranted" (Lines 552–556).

12. Recommendation based on finding, be carefully about this

**Response from authors:** Thank you for the comment. We have revised our recommendations to ensure that they are based on our findings. Recommendations now directly reflect key results:Gender-specific screening protocols (aligning with sex-based risk differences); Socioeconomic programs targeting education/employment (leveraging education’s protective effect); Family-centered interventions (supporting marriage’s protective role).; Enhanced addiction services for frequently hospitalized patients.

13. Is it ASSIT valid in Zambia? If not valid it is better to recommend future study to do validation.

**Response from authors:** Thank you so much. We note that cultural validity of ASSIST in Zambia (e.g., for kachasu or traditional substances) requires confirmation. We have revised to recommend future validation studies adapting ASSIST to Zambian contexts. We acknowledge this gap: "ASSIST’s cultural validity for local substances (e.g., kachasu) remains unconfirmed; future studies should validate adapted tools" (Lines 540–542).

**Reviewer 2**
1. Clearly define "substance abuse" to align with ICD-10, including alcohol (F10) and nicotine (F17). Ensure consistency across methodology and results to avoid confusion.

**Response from authors:** Thank so much. We have revised and defined substance use in the methodology to align with ICD-10.

2. Include a power calculation to justify the sample size of 303 participants. This is necessary to confirm the study’s ability to detect associations. Eg for rare variables like suicidal behavior (n=3).

**Response from authors:** Thank you for the guidance. We have revised and included power calculation in the methodology to justify the sample size. A power calculation was conducted using G*Power 3.1. With an expected substance use prevalence of 50% (based on SSA literature), α=0.05, power=80%, and margin of error=5%, a minimum sample of 246 was required. Our sample (N=303) exceeds this threshold. However, subgroup analyses (e.g., suicidality) were underpowered due to low frequencies. We added: "Post-hoc power analysis (G*Power 3.1) showed 99.8% power to detect the primary predictor (male sex) effect (OR=6.79). For rare outcomes (e.g., suicidality, n=3), power was 12%—highlighting analytical limitations for infrequent variables" (Lines 128–132).

3. Analyze the 538 excluded records (64% of 840) to assess systematic differences from included cases. High exclusion rates may introduce bias.

**Response from authors:** We appreciate the reviewer’s insightful comment regarding the potential for bias introduced by the exclusion of 538 records. Unfortunately, we are unable to conduct additional analyses on these records, as they were excluded due to the absence of key outcome variables and other critical data necessary for our analysis. These records lacked essential information which made them unsuitable for inclusion in the study.

We acknowledge the potential for selection bias and have addressed this limitation in the revised discussion section. Nonetheless, the decision to exclude these cases was based on methodological rigor and the need to ensure valid comparisons and robust conclusions using complete and analyzable data.

We clarify: "Excluded records lacked core variables (e.g., substance use status), precluding comparative analysis. This introduces potential selection bias, acknowledged as a limitation" (Lines 173–175 and 534–535).

4. Provide details on the quality and consistency of medical record documentation. Specify any quality checks or standardization processes applied.

**Response from authors:** Thank you so much for the guidance. We have revised and included details on the quality and consistency of medical documentation. Data quality was ensured through: pre-defined abstraction criteria using a standardized REDCap form, dual independent extraction for 10% of records to verify consistency and exclusion of records with >20% missing core variables (e.g., substance use status, diagnosis)

5. State the criteria for selecting variables for multivariable logistic regression, such as p<0.05 in univariable analysis or theoretical relevance.

**Response from authors:** We detail our approach: "Variables were selected via backward elimination (retaining *p* < 0.05), clinical relevance, VIF < 10, |*r*| < 0.8, and ≥10 events per predictor. Model fit was assessed via AIC, BIC, and Hosmer-Lemeshow tests" (Lines 222–228).

Variable selection for multivariable logistic regression required balancing statistical rigor with clinical relevance and practical constraints. Key steps included an initial screening phase using liberal p-values (e.g., < 0.20 - 0.25), followed by final variable inclusion based on stricter statistical significance (p < 0.05). Crucially, clinically important variables were mandatorily considered for inclusion regardless of their statistical p-value. Potential multicollinearity among predictors was assessed using the Variance Inflation Factor (VIF < 10), absolute correlation coefficients (|r| < 0.8), and condition index (< 30). Sample size sufficiency was ensured, ideally targeting a minimum of 10-15+ events per predictor variable. Various selection methods (e.g., Forward, Backward, Stepwise, Best Subset) were employed, guided by criteria such as p-values or information criteria like the Akaike Information Criterion (AIC) and the Bayesian Information Criterion (BIC).

Model comparison and refinement utilized indices including AIC, BIC, the Area Under the receiver operating characteristic Curve (AUC), and goodness-of-fit tests such as the Hosmer-Lemeshow test. Practical considerations, such as data availability, cost, and interpretability, were integrated alongside the evaluation of potential interaction terms. Thorough validation, encompassing both internal and external approaches, was deemed critical for developing a robust, generalizable, and clinically useful model.

This comprehensive framework guided our approach in the schizophrenia study. Specifically, we applied backward elimination (starting with all candidate variables and sequentially removing those with p > 0.05), compared competing models based on AIC values, and assessed goodness-of-fit using the Hosmer-Lemeshow test. This process resulted in a final, robust 7-variable model with optimized performance

6. Assess collinearity between predictors, such as alcohol use and smoking, using variance inflation factor (VIF) or correlation matrices. Report goodness-of-fit with tests like Hosmer-Lemeshow.

**Response from authors:**

**We assessed collinearity between predictors by first performing a correlation matrix as shown below**

**High correlation pairs (|r| > 0.7): The correlation matrix shows no high correlations (>0.7) between predictors, which is good for avoiding multicollinearity. We then calculated the Variance Inflation Factor (VIF) for a more precise assessment of collinearity and removed the variables with highest VIF and performed a logistic regression modeling to predict current substance abuse.**

Logistic Regression Model Results:

**Hosmer-Lemeshow Goodness-of-Fit Test**

Chi-square statistic: 8.5747; Degrees of freedom: 8; P-value: 0.3794

Result: Good fit (p > 0.05, fail to reject null hypothesis)

Significant Predictors (p < 0.05):

## Key Findings:

**Model Fit**: The Hosmer-Lemeshow test indicates good model fit (p = 0.3794 > 0.05), meaning we fail to reject the null hypothesis of adequate fit.

**Significant Predictors** (p < 0.05):

- **Alcohol use**: Strongest predictor (OR = 8.78) - patients with alcohol use are nearly 9 times more likely to have current substance abuse
- **Smoking**: Second strongest (OR = 5.03) - smokers are 5 times more likely to have substance abuse
- **Hallucinations**: Increases odds by 3 times (OR = 3.00)
- **Sex**: Males have lower odds (OR = 0.27) - being female increases substance abuse risk
- **Both parents deceased**: Protective factor (OR = 0.18) - surprisingly reduces substance abuse risk
- **Education**: Higher education is protective (OR = 0.45)

**Model Performance**: The model shows good discriminative ability with 83% accuracy, pseudo R² of 0.46, and reasonable precision/recall for both classes.

We then performed model comparison to use the best model for our multivariable analysis as shown below:

As shown, Best model based on AIC is Backward Elimination. We also performed Cross-Validation comparison and the Cross-Validation Results (5-fold) are shown below:

The final multivariable analysis model is shown below using the best model (backward elimination method)

Hosmer-Lemeshow Test Results:

Chi-square statistic:18.2212

Degrees of freedom:8

P-value:0.0196; Poor fit (p <= 0.05)

The analysis shows that alcohol use and smoking are the strongest predictors of current substance abuse, with odds ratios of 6.09 and 4.80 respectively. Interestingly, several factors appear protective, including being male, married, having higher education, and having both parents deceased. The backward elimination model performed best overall with the lowest AIC, though the Hosmer-Lemeshow test suggests some calibration issues that might be addressed with additional model refinements.

In summary We state: "No pairwise correlations exceeded |*r*| > 0.7. The final model showed good fit (Hosmer-Lemeshow *p* = 0.379) and discrimination (AUC = 0.83)" (Lines 312–314 and Supplementary Table 2). We thank the reviewer for pointing out as it has helped in improving clarity of results and interpreting results in context.

7. Explain wide confidence intervals, such as for male sex (AOR=5.21, 95% CI: 1.75–15.63).

**Response from authors:** Thank you for the comment. We have revised and included an explanation for the wide intervals in our discussion. Wide confidence intervals for male sex (AOR=5.21, 95% CI: 1.75–15.63) reflect limited female substance users (n=13), reducing precision. Despite this, the strong effect size (AOR>5) and statistical significance (p=0.003) support male gender as a key predictor.

8. Limit the scope of results to tertiary settings similar to LUTH. From a single-center study, this cannot be claimed as “nationally representative data”.

**Response from author:** Thank you for the guidance. We have revised and included a statement to indicate that the findings reflect tertiary care patients at LUTH. For that reason, we have also highlighted is as a limitation and that may not generalize to community settings or regions with limited healthcare access. We revised: "Findings reflect patients at Zambia’s largest referral hospital and may not generalize to community settings" (Lines 542–543).

**Reviewer 3**
1. The authors should include a sub-section on the aim or objectives of the study just after the background. Authors should also include the summary of data analysis plan in the methods.

**Response from author:** Thank you for the comment. We have revised and included objectives at the end of the introduction. Objectives were added to the Introduction: "This study aimed to determine prevalence and correlates of substance use among adults with schizophrenia at LUTH" (Lines 78–79). The Methods now summarize analysis: "Univariable/multivariable logistic regression identified factors associated with substance use" (Lines 215–216).

2. In the line 66, the authors should insert Introduction instead of Background. The authors should incorporate the objective(s) of the study at the end of the sub-section

**Response from author:** Thank you for the guidance. We have revised and replaced background with introduction.

3. The study design is not clear and the authors should think through it since in the abstract it is a different design. The authors should include a sub-section on the study population i.e. target population, accessible population and the actual study population recruited in the study just before eligibility and recruitment. Furthermore, the study populations are schizophrenic patients and they have lost reality of the world and therefore the responses they give may not be well connected. Which type of schizophrenic patients’ records were used or participated in the study? In the abstract, data was collected using structured interviews, validated tools (Alcohol, Smoking, and Substance Involvement Screening Test [ASSIST]), but in the methods medical records were used, how were interviews made on the records?

**Response from author:** Thank you for the comment. We have revised and included sub-section under study population to include target population, accessible population and actual population. Additionally, we have revise to indicate that Data were collected from medical records including records of structured interviews with validated tools (Alcohol, Smoking, and Substance Involvement Screening Test [ASSIST], Positive and Negative Syndrome Scale [PANSS]). No interviews were conducted in this study but we utilized records of structured interviews which part of medical records. We specify: "This retrospective cross-sectional study utilized medical records containing structured ASSIST/PANSS assessments from clinician interviews. All schizophrenia subtypes (ICD-10: F20.0–F20.9) were included" (Lines 108–110, 137–139).

4. In table 1, demographic characteristics, the authors should include the units of age, and since the main measure is % and not frequencies, the authors should use % (n) and not the reverse. The total number of participants was 302, but in table 1, some items responses exceed that number. The authors should check this throughout the table. In table2, there is a strong association of Violent Behavior and substance use, how do violent behaviors lead to substance abuse?

**Response from author:** Thank you so much. We have revised and included the units for age and changed the main measure to %. We have also double checked our dataset which we used for analysis and confirmed that the number of participants was 303, not 302 and have since changed it. The findings on the relationship between violent behavior and substance use in schizophrenia is backed by neuropsychiatric evidence that the relationship involves bidirectional pathways influenced by biological, psychological, and contextual factors. Schizophrenia impairs prefrontal cortex function, reducing inhibitory control over aggression and reward-seeking. Substance use exacerbates this by dysregulating dopamine/glutamate systems. Furthermore, psychotic symptoms (e.g., persecutory delusions) may trigger aggression; substances like alcohol temporarily reduce distress but increase disinhibition. However, violent behavior lost significance in the multivariable analysis. This may indicate that violence may be an outcome of substance use (e.g., alcohol-induced disinhibition) rather than a primary cause.

5. The authors should split the discussion if possible based on the study objectives so that it is clear i.e. demographic characteristics, substance use and associated risk factors.

**Response from author:** thank you. The Discussion now follows: Prevalence and comparisons (Lines 396–407); Risk/protective factors (Lines 408–461); Clinical implications (Lines 462–503).

6. The authors should have a sub-section of conclusions in the manuscript and not entangled in the discussion. And the conclusion made neither is nor supported by the findings of the study and therefore it needs to be re-written.

**Response from author:** Thank you for the comment. We have revised and included a section for conclusion and re-written it in alignment with the findings. A standalone "Conclusion" section was added (Lines 552–559), summarizing key findings and recommendations.

6. Further citations are needed to be included to support the background to the study and the authors should also follow the recommended Journal reference style.

**Response from author:**	We added additional citations (e.g., Nigerian/Ethiopian prevalence studies) and verified reference formatting per journal guidelines.

Yours,

Prof Sepiso K. Masenga

On behalf of the authors
