# Supplementary material for: Prevalence and factors associated with illicit substance use among persons with Schizophrenia at a Tertiary Referral Hospital in Zambia
Source: PLOS Glob Public Health. 2026 Jan 12;6(1):e0005024. doi: 10.1371/journal.pgph.0005024 (PMC12795356; doi:10.1371/journal.pgph.0005024)
Supplement: Attachment — Submitted filename: Schizophrenia Manuscript track changes last review.docx [file pgph.0005024.s009.docx]

**Prevalence and Factors Associated with Substance use Among Persons with Schizophrenia at a Tertiary Referral Hospital in Zambia**

Kimberley R. Kurehwatira^1,2#^, Emmanuel L. Luwaya^1,2#^, Joreen P. Povia^3^, Chileleko Siakabanze^2^, Salma M. Baines^1,2^, Emmanuel Yumba^1,2^, Prince Mulambo^1,2^, David N. Masta^1,2^, Nestorine N. Ngongo^1,2^, Natasha Chishala^1,2^, Emmanuel O. Riwo^1,2^, Katongo H. Mutengo^2^, Hanzooma Hatwiko^1,2^, Martin Chakulya^1,2^, Lukundo Siame^1,2^, Bislom C. Mweene^1,2^, Sepiso K. Masenga^1,2*^

^1^Department of Pathology, Mulungushi University, School of Medicine and Health Sciences, Livingstone, Zambia

^2^Department of Pathology, Livingstone Center for Prevention and Translational Science, Livingstone, Zambia

^3^Department of Health Economics, Livingstone Center for Prevention and Translational Science, Livingstone, Zambia

***Corresponding author:** [sepisomasenga@gmail.com](mailto:sepisomasenga@gmail.com) or [sepisomasenga@lcpts.org](mailto:sepisomasenga@lcpts.org)

#share co-first authorship

**Abstract**

**Introduction**

Schizophrenia frequently involves comorbid substance use, exacerbating symptoms and reducing treatment efficacy. In Zambia, systemic challenges like poverty and fragmented healthcare amplify this burden, yet no prior studies have examined this comorbidity locally. This study aimed to determine the prevalence and factors associated with substance use among adults with schizophrenia at the University Teaching Hospital (LUTH).

**Methods**

A hospital-based single center cross-sectional study was conducted at LUTH, Zambia’s largest referral center, from January to June 2023. A sample of 303 adults with schizophrenia were recruited via systematic random sampling. Data were collected from existing medical records which included documentation from structured interviews with validated tools (Alcohol, Smoking, and Substance Involvement Screening Test [ASSIST], Positive and Negative Syndrome Scale [PANSS]) conducted as part of routine clinical care. Sociodemographic, clinical, and substance use variables were analyzed using chi-square tests and multivariate logistic regression to identify factors associated with substance use.

**Results**

The prevalence of substance use was 31.1% (94/303). Alcohol use (AOR = 6.08, 95% CI: 3.14–11.78, p< 0.0001) and tobacco smoking (AOR = 4.80, 95% CI: 2.44–9.46, p< 0.0001) were strongly associated with substance use. Factors associated with lower odds of substance use included female sex (AOR = 0.27, 95% CI: 0.12–0.60, p= 0.001), marriage (AOR = 0.39, 95% CI: 0.19–0.79, p= 0.008), having both parents deceased (AOR = 0.29, 95% CI: 0.11–0.75, p= 0.011), and higher education (AOR = 0.44, 95% CI: 0.24–0.81, p= 0.009). Increased hospitalizations were associated with substance use (AOR = 1.29, 95% CI: 1.01–1.65, p= 0.038).

**Conclusion**

This first Zambian study found a substance use prevalence of 31.1% among persons with schizophrenia. The findings reveal associations between male gender, alcohol use, and tobacco smoking and substance use, while female sex, marriage, parental loss, and education were associated with lower odds of use. Frequent hospitalizations were also associated with substance use. Integrated screening, gender-specific interventions, and socioeconomic support are urgently needed in Zambia’s resource-constrained setting.

**Key Words:** Substance use; Schizophrenia; Zambia; Disorganized Behaviour; Disorganized speech; violent behaviour; hallucinations; associated factors; alcohol use; tobacco smoking; dual diagnosis, LCPTS; Livingstone Center for Prevention and Translational Science

**Introduction**

Schizophrenia, a debilitating psychiatric disorder affecting approximately 24 million people globally (WHO, 2022) [1], is characterized by profound disruptions in cognition, emotion, and behaviour [2]. In sub-Saharan Africa (SSA), schizophrenia is compounded by systemic challenges such as poverty, stigma, and fragmented healthcare systems. Comorbid substance use, prevalent in 40–60% of schizophrenia cases globally [3], exacerbates disease burden, impairing treatment adherence and increasing relapse rates. In SSA, this comorbidity is particularly severe, with studies reporting substance use rates as high as 58–65% among persons with schizophrenia [4]. Zambia, a low-income country with a population of 22 million, faces acute mental health challenges: mental disorders account for 13% of the national disease burden, yet the country has only ten psychiatrists as reported by WHO in 2023 [5], a slight increase from five psychiatrists in 2017, and limited mental health funding [6]. The University Teaching Hospital (LUTH), Zambia’s largest referral center, struggles with high patient loads and resource constraints, making it a critical site for studying schizophrenia and substance use.

Substance use patterns are shaped by cultural, economic, and environmental factors [7–12]. The widespread use of *kachasu* (a cheap, a traditionally fermented, highly intoxicating, distilled alcoholic spirit), cannabis, and emerging synthetic drugs is normalized in many communities. Economic hardship affecting 52.4% of Zambians who live below the poverty line [13] and unemployment (13.1% nationally) drive maladaptive coping behaviours, including substance use. Traditional beliefs attributing mental illness to spiritual causes further delay biomedical care, pushing patients toward self-medication with substances [10]. Despite these challenges, no studies have systematically examined the prevalence or correlates of substance use among persons with schizophrenia in Zambia. This gap impedes the development of culturally tailored interventions, perpetuating cycles of poor outcomes.

Zambia’s mental health system is strained: mental health receives <1% of the health budget, and community stigma forces many patients into isolation [13]. Substance use among persons with schizophrenia at LUTH remains unstudied, yet it likely fuels high relapse rates and recurrent hospitalizations. This study aimed to determine the prevalence of substance use and identify sociodemographic and clinical factors associated with it among persons with schizophrenia at LUTH. We hypothesized that male sex, alcohol use, and tobacco smoking would be associated with higher odds of substance use, while factors like higher education and being married would be associated with lower odds.

**Methodology**

**Ethics Statement**

Ethical approval was obtained from the Mulungushi University School of Medicine and Health Sciences Research Ethics Committee (ethics reference number SMHS-MU1-2025-42) on 24 March 2025. Administrative permission was granted by LUTH management. All extracted data were anonymized to protect confidentiality. All data collected and analyzed were de-identified to ensure complete confidentiality. No information leading to identification of patients during and after analysis was abstracted and entered in the data collection form. Secondary data were used in this project. A written/verbal consent was not applicable and was therefore waived by the ethics committee.

**Study Design**

This was a retrospective cross-sectional study. We used existing medical records from persons with schizophrenia at LUTH in Zambia.

**Study Setting**

The research was conducted at the Psychiatry Department of LUTH, a tertiary referral hospital located in Livingstone, Zambia. The department systematically documents clinical features, substance use history, and treatment of persons diagnosed with schizophrenia.

**Study Population**

*Target Population*

Adults (≥18 years) with schizophrenia in Livingstone.

*Accessible Population*

Persons with Schizophrenia seeking care at LUTH’s Psychiatry Department (between January 2021 and December 2024).

**Actual Study Population**

303 patients meeting inclusion criteria, representing 36.1% of screened records (303/840). All subtypes of schizophrenia (ICD-10: F20.0–F20.9) were included.

A post-hoc power analysis was conducted using G*Power 3.1 to evaluate the statistical power of our study design. This analysis revealed 99.8% power at α=0.05 to detect the observed effect size (OR=6.79) for the primary variable of male sex. However, for rare outcomes such as suicidality, where only three cases were observed, the power was substantially lower at just 12% to detect meaningful associations. The power calculation for male sex specifically employed a two-proportion z-test for independent samples with a two-tailed design. This test compared proportions of 0.448 (substance use in males) and 0.107 (substance use in females), with sample sizes of 181 males and 122 females at the conventional alpha level of 0.05. These parameters confirmed robust power for our main sex-based comparison while highlighting limitations for analyzing infrequent outcomes.

**Eligibility and Recruitment**

We reviewed medical records of persons diagnosed with schizophrenia who received care at LUTH between January 2021 and December 2024. The diagnosis of schizophrenia was made by a consultant psychiatrist according to ICD-10 criteria. Both inpatients and outpatients were included, regardless of their clinical remission status. Records were included if the patient was 18 years or older, had a confirmed psychiatrist-diagnosed schizophrenia (ICD-10 F20), and the medical record contained complete documentation of sociodemographic data and substance use history. A total of 303 patient records were included in the final analysis. Records were included if the patient had a confirmed diagnosis of schizophrenia and complete documentation of sociodemographic and substance use information. Records missing key variables were excluded.

**Data Collection**

Data were collected through a structured file review process between 1^st^ April and 28 April 2025. Trained research assistants extracted relevant data from patients’ medical files into a REDCap database. No patient interviews were conducted. Information from structured clinical interviews (e.g., ASSIST, PANSS) had been previously conducted by clinic staff and documented in the records; this historical data was extracted for analysis. To ensure data quality, 10% of the records were subjected to dual review for accuracy and consistency, and performed an audit trail using RedCap audit logs to track all data entries and revisions. Additionally, records missing variables were excluded. Other quality measures included the training of personnel involved in data collection using mock records and piloting on records to refine variable definitions.

**Assessment of Clinical Variables**

Clinical variables were ascertained from the psychiatrist's notes and assessment scales documented in the medical records. Hallucinations, delusions, disorganized speech, disorganized behavior, and negative symptoms were assessed and documented by the treating psychiatrist based on clinical evaluation and, where available, PANSS scores. Cognitive impairment was noted based on clinical observation and any documented cognitive assessments. Suicidality and self-harm were defined by documented reports of suicidal ideation, plans, or attempts, or acts of non-suicidal self-injury. Violent behavior was defined as documented episodes of physical aggression towards others or property.

**Study Variables**

The primary outcome variable was current substance use, defined as documented use of psychoactive substances (cannabis, cocaine, opioids, or others) within the past 12 months.
Independent variables included: Sociodemographic Variables (Age, Sex, Marital status, Educational level, Employment status, Residence, Parental status) and Clinical Variables (hallucinations and type, delusions and type, disorganized speech, disorganized behavior, negative symptoms, cognitive impairment, suicidality and self-harm, violent behavior, family history, tobacco smoking status, awareness of diagnosis, consultation with traditional healers, medication adherence, number of hospitalizations, hematological and biochemical parameters).

**Data Analysis**

Data were exported from REDCap into Microsoft Excel 2013 for cleaning and coding, then analyzed using StatCrunch. Descriptive statistics summarized categorical variables (frequencies and percentages) and continuous variables (medians and interquartile ranges [IQR]). Normality was assessed using the Shapiro-Wilk test. Associations between categorical variables were evaluated using the Chi-square test, while differences between continuous variables were assessed using the Wilcoxon rank-sum test. Univariable and multivariable logistic regression analyses were performed to identify factors associated with current substance use. Adjusted odds ratios (AORs) with 95% confidence intervals (CIs) were reported.

Variable selection for multivariable logistic regression required balancing statistical rigor with clinical relevance and practical constraints. Key steps included an initial screening phase using liberal p-values (e.g., < 0.20 - 0.25), followed by final variable inclusion based on stricter statistical significance (p < 0.05). Crucially, clinically important variables were mandatorily considered for inclusion regardless of their statistical p-value. Potential multicollinearity among variables was assessed using the Variance Inflation Factor (VIF < 10), absolute correlation coefficients (|r| < 0.8), and condition index (< 30). Sample size sufficiency was ensured, ideally targeting a minimum of 10-15+ events per variable. Various selection methods (e.g., Forward, Backward, Stepwise, Best Subset) were employed, guided by criteria such as p-values or information criteria like the Akaike Information Criterion (AIC) and the Bayesian Information Criterion (BIC).

Model comparison and refinement utilized indices including AIC, BIC, the Area Under the receiver operating characteristic Curve (AUC), and goodness-of-fit tests such as the Hosmer-Lemeshow test. Practical considerations, such as data availability, cost, and interpretability, were integrated alongside the evaluation of potential interaction terms. Thorough validation, encompassing both internal and external approaches, was deemed critical for developing a robust, generalizable, and clinically useful model.

This comprehensive framework guided our approach in the schizophrenia study. Specifically, we applied backward elimination (starting with all candidate variables and sequentially removing those with p > 0.05), compared competing models based on AIC values, and assessed goodness-of-fit using the Hosmer-Lemeshow test. This process resulted in a final, robust 7-variable model with optimized performance.

We used the Strengthening the Reporting of Observational Studies in Epidemiology to guide the writing (Supplementary file S1.)

**Results**

From a total of 1000 files available for abstraction, 840 medical records were reviewed, 303 records met the inclusion criteria and were included in the analysis. The remaining 538 records were excluded due to incomplete information, such as missing details on age, sex, residence, diagnosis or the outcome, **Fig 1.**

**Figure 1. Eligibility flow diagram**

**Characteristics of the study population**

A total of 303 participants were enrolled in this study, with a median age of 31 years (IQR: 24–41), **Table 1.** The cohort comprised 59.7% males (n=181) and 40.3% females (n=122). Bivariate analyses revealed numerous significant associations with substance use. Females exhibited substantially lower substance use prevalence than males (10.7% vs. 44.8%, p<0.0001). Married participants had significantly lower substance use rates compared to unmarried individuals (17.3% vs. 43.2%, p<0.0001). Those with both parents deceased showed reduced substance use (18.0% vs. 34.9% in others, p=0.011), while participants with negative symptoms had lower rates than those without (19.2% vs. 33.9%, p=0.038). Substance use was strongly associated with alcohol use (60.2% vs. 13.7% in non-users, p<0.0001), tobacco smoking (61.5% vs. 15.1% in non-smokers, p<0.0001), and violent behavior (67.0% vs. 38.5% in non-violent participants, p<0.0001). Medication non-adherence was linked to higher substance use (46.0% vs. 16.4% in adherent individuals, p<0.0001), while awareness of one's condition was associated with lower substance use (21.2% vs. 39.5% in unaware individuals, p=0.0006). The substance use group had significantly more hospitalizations (median=3, IQR:2–4) than the non-use group (median=2, IQR:1–3, p=0.044), and younger age was associated with substance use (p=0.023). Participants with suicidal ideation (n=3) all had substance use (p=0.029).

| **Table 1. Characteristics of the study populations** | | | | |
| --- | --- | --- | --- | --- |
|  |  | *Substance use % (n)* | |  |
| *Variable* | *Median (IQR) or Frequency (%)* | *No* | Yes | P. value |
| *Age (years)* | 31 (24,41) | 68.9 (208) | 31.1 ( 94) | **0.023** |
| *Sex, n=301* |  |  |  |  |
| *Male* | *59.7 (181)* | 55.3 (100) | 44.8 (81) | **<0.0001** |
| *Female* | *40.3 (122)* | 89.3 (109) | 10.7 (13) |  |
| *Marital Status, n=301* |  |  |  |  |
| *Married* | *46.2 (139)* | 82.7 (115) | 17.3 (24) |  |
| *Not Married* | *53.8 (162)* | 56.8 (92) | 43.2 (70) | **<0.0001** |
| *Educated, n=300* |  |  |  |  |
| *Primary* | *47.7 (143)* | 63.6 (91) | 36.4 (52) | 0.074 |
| *Secondary* | *46.7 (140)* | 71.4 (100) | 28.6 (40) |  |
| *Tertiary* | *5.7 (17)* | 88.2 (15) | 11.8 (2) |  |
| *Employment Status, n=292* |  |  |  |  |
| *Unemployed* | *69.6 (204)* | 66.2 (135) | 33.8 (69) | 0.080 |
| *Employed* | *30.4 (88)* | 76.4 (68) | 23.6 (21) |  |
| *Residence, n=297* |  |  |  |  |
| *Urban* | *82.8 (246)* | 68.7 (169) | 31.3 (77) | 0.587 |
| *Rural* | *17.17 (51)* | 72.6 (37) | 27.4 (14) |  |
| *Both Parents Alive, n=296* |  |  |  |  |
| *No* | *58.8 (174)* | 70.7 (123) | 29.3 (51) | 0.431 |
| *Yes* | *41.2 (122)* | 66.4 (81) | 33.6 (41) |  |
| *Both Parents Deceased, n=296* |  |  |  |  |
| *No* | *79.4 (235)* | 65.1 (153) | 34.9 (82) | **0.011** |
| *Yes* | *20.6 (61)* | *82 (50)* | 18 (11) |  |
| *Hallucinations, n=301* |  |  |  |  |
| *No* | *18.3 (55)* | *69.1 (38)* | 30.9 (17) | 0.954 |
| *Yes* | *81.7 (246)* | *68.7 (169)* | 31.3 (77) |  |
| *Delusions,* |  |  |  |  |
| *No* | *41.9 (127)* | *69.3 (88)* | 30.7 (39) | 0.919 |
| *Yes* | *58.1 (176)* | *68.7 (121)* | 31.3 (55) |  |
| Disorganized Speech, *n=300* |  |  |  |  |
| *No* | 43.7 (131) | 74 (97) | 26 (34) | 0.096 |
| *Yes* | 56.3 (169) | 65.1 (207) | 34.9 (59) |  |
| Disorganized Behaviour, *n=302* |  |  |  |  |
| No | 8.3 (25) | 72 (18) | 28 (7) | 0.724 |
| Yes | 91.7 (277) | 68.6 (190) | 31.4 (87) |  |
| Negative Symptoms, *n=300* |  |  |  |  |
| *No* | 82.7 (248) | *66.1 (164)* | *33.9 (84)* | ***0.038*** |
| *Yes* | 17.3 (52) | *80.8 (42)* | *19.2 (10)* |  |
| Cognitive Impairment, *n=300* |  |  |  |  |
| *No* | 39 (117) | *75.2 (88)* | *24.8 (29)* | *0.062* |
| *Yes* | 61 (183) | *65 (119)* | *35 (64)* |  |
| Suicidal, |  |  |  |  |
| *No* | 99.0 (300) | *69.7 (209)* | *30.3 (91)* | ***0.029*** |
| *Yes* | 0.1 (3) | *0* | *100 (3)* |  |
| History of Self Harm, *n=302* |  |  |  |  |
| *No* | 93 (281) | *68.7 (193)* | *31.3 (88)* | *0.793* |
| *Yes* | 7 (21) | *71.4 (15)* | *28.6 (6)* |  |
| Family History of Schizophrenia |  |  |  |  |
| *No* | 97 (294) | *68.7 (202)* | *31.3 (92)* | *0.725* |
| *Yes* | 3 (9) | *77.8 (7)* | *22.2 (2)* |  |
| Family History of Other Psychiatric Illness, *n=294* |  |  |  |  |
| *No* | 75.3 (225) | *71.6 (161)* | *28.4 (64)* | *0.128* |
| *Yes* | 24.7 (74) | *62.1 (46)* | *37.9 (28)* |  |
| Alcohol Use |  |  |  |  |
| *No* | 62.7 (190) | *86.3 (164)* | *13.7 (26)* | ***<0.0001*** |
| *Yes* | 37.3 (113) | *39.8 (45)* | *60.2 (68)* |  |
| Violent Behaviour, *n=302* |  |  |  |  |
| *No* | 52.6 (159) | 61.5 (128) | 38.5 (80) | **<0.0001** |
| *Yes* | 47.4 (143) | 33 (31) | 67 (63) |  |
| Tobacco Smoking, *n=302* |  |  |  |  |
| *No* | 65.6 (198) | 84.9 (168) | 15.1 (30) | **<0.0001** |
| *Yes* | 34.4 (104) | 38.5 (40) | 61.5 (64) |  |
| Aware Of Condition, *n=299* |  |  |  |  |
| *No* | 54.2 (162) | *60.49 (98)* | *39.5 (64)* | ***0.0006*** |
| *Yes* | 45.8 (137) | *78.8 (108)* | *21.2 (29)* |  |
| Consulted Traditional Healers, *n=241* |  |  |  |  |
| No | 83.0 (200) | 68 (136) | 32 (64) | 0.514 |
| Yes | 17.0 (41) | 73.2 (30) | 26.8 (11) |  |
| Patient Adherent to Medication, *n=293* |  |  |  |  |
| *No* | 48.1 (141) | *54 (76)* | *46 (65)* | ***<0.0001*** |
| *Yes* | 51.9 (152) | *83.6 (127)* | *16.4 (25)* |  |
| Number of hospitalizations | 2 (2, 3) | 2 (1, 3) | 3 (2, 4) | **0.044** |

**Assessment of collinearity between variables**

Firstly, the correlation matrix performed showed no high correlations (>0.7) between variables, which is good for avoiding multicollinearity, **Fig 2.**

**Figure 2. Correlation matrix of variables**

For a more precise assessment of collinearity, we calculated the Variance Inflation Factor (VIF) **(supplementary Table 1)** for fitting a logistic regression model and performed the Hosmer-Lemeshow goodness-of-fit test. The Hosmer-Lemeshow test indicated a good model fit (p = 0.3794 > 0.05), meaning we fail to reject the null hypothesis of adequate fit, **Supplementary Table 2.** The model showed good discriminative ability with 83% accuracy, pseudo R² of 0.46, and reasonable precision/recall for both classes.

**Factors associated with Substance use in Logistic Regression**

We performed a model comparison and cross-validation comparison for the best logistic regression model for the variables in the study. The backward elimination model performed best overall with the lowest AIC, though the Hosmer-Lemeshow test (Pseudo R-squared of 0.3863; AIC of 246.33 (lowest among all models) and Cross-validation accuracy of 75.93%) suggested poor calibration (p = 0.0196) that might be addressed with additional model refinements, **Supplementary Table 3**.

The analysis revealed several factors significantly associated with substance use, both in the initial unadjusted models (OR) and after adjusting for other variables (AOR), **Table 2**. Sex demonstrated a strong protective effect. In the unadjusted model, the odds of substance use were significantly lower for females compared to males (OR = 0.14, 95% CI: 0.07-0.28, p < 0.0001). This association remained significant, though attenuated, in the adjusted model (AOR = 0.27, 95% CI: 0.12-0.60, p = 0.001). Similarly, being Married was associated with substantially lower odds of substance use initially (OR = 0.27, 95% CI: 0.16-0.47, p < 0.0001), and this association persisted even after adjustment (AOR = 0.39, 95% CI: 0.19-0.79, p = 0.008).

The status of having Both Parents Deceased also emerged as associated with lower odds of substance use. The unadjusted model showed lower odds (OR = 0.41, 95% CI: 0.20-0.83, p = 0.013), and interestingly, this association became stronger in the adjusted model (AOR = 0.29, 95% CI: 0.11-0.75, p = 0.011). Higher levels of being Educated were associated with moderately lower odds of substance use in the unadjusted analysis (OR = 0.62, 95% CI: 0.40-0.95, p = 0.029). After adjustment, this association increased in magnitude and significance (AOR = 0.44, 95% CI: 0.24-0.81, p = 0.009).

Conversely, Alcohol Use was strongly associated with substance use. Individuals using alcohol had dramatically higher odds of substance use in the unadjusted model (OR = 9.53, 95% CI: 5.44-16.67, p < 0.0001). Although this association lessened somewhat in the adjusted model, it remained highly significant and strong (AOR = 6.08, 95% CI: 3.14-11.78, p < 0.0001). Tobacco smoking exhibited a similarly strong association. The unadjusted odds were very high (OR = 8.96, 95% CI: 5.15-15.59, p < 0.0001), and while reduced, the association remained large and highly significant after adjustment (AOR = 4.80, 95% CI: 2.44-9.46, p < 0.0001).

The Number of hospitalizations showed a trend towards being associated with substance use in the unadjusted model but did not reach conventional statistical significance (OR = 1.16, 95% CI: 0.98-1.38, p = 0.076). However, after adjusting for other factors, a higher number of hospitalizations became a statistically significant variable associated with increased odds of substance use (AOR = 1.29, 95% CI: 1.01-1.65, p = 0.038).

Several factors were significant in the unadjusted model but were not retained or reported in the final adjusted model presented. Exhibiting Violent Behaviour was associated with substantially higher odds of substance use (OR = 3.25, 95% CI: 1.94-5.43, p < 0.0001). Higher levels of Negative Symptoms appeared to have an association with lower odds of substance use (OR = 0.46, 95% CI: 0.22-0.97, p = 0.041). Being Aware of Condition was associated with significantly lower odds of substance use (OR = 0.41, 95% CI: 0.25-0.69, p = 0.0008). Adherence to medication was associated with lower odds of substance use in the unadjusted analysis (OR = 0.23, 95% CI: 0.134-0.39, p < 0.0001). Finally, increasing Age was associated with slightly lower odds of substance use in the unadjusted model (OR = 0.97, 95% CI: 0.95-0.99, p = 0.020).

In summary, the variables most strongly associated with substance use in the adjusted model were alcohol use and tobacco smoking. Variables associated with lower odds of substance use in the adjusted model included being female, being married, having both parents deceased, and higher education. An increased number of hospitalizations was also associated with substance use after adjustment. Factors like violent behaviour, negative symptoms, awareness of condition, medication adherence, and age were significant in the bivariate analysis but were not included in the final adjusted model (did not retain significance and did not meet the criteria for inclusion in the final model).

| **Table 2. Logistic regression analysis for Substance use** | | | | |
| --- | --- | --- | --- | --- |
| **Variables** | **OR (95%CI)** | **p. value** | **AOR (95%CI)** | **p. value** |
| Sex | 0.14 (0.07,0.28) | **<0.0001** | 0.27 (0.12, 0.60) | **0.001** |
| Married | 0.27 (0.16,0.47) | <**0.0001** | 0.39 (0.19, 0.79) | **0.008** |
| Both Parents Deceased | 0.41 (0.20,0.83) | **0.013** | 0.29 (0.11,0.75) | **0.011** |
| Educated | 0.62 (0.40, 0.95) | **0.029** | 0.44 (0.24, 0.81) | **0.009** |
| Alcohol Use | 9.53 (5.44,16.67) | **<0.0001** | 6.08 (3.14,11.78) | **<0.0001** |
| Smoking | 8.96 (5.15,15.59) | **<0.0001** | 4.80 (2.44,9.46) | **<0.0001** |
| Number of hospitalizations | 1.16 (0.98,1.38) | 0.076 | 1.29 (1.01,1.65) | **0.038** |
| Violent Behaviour | 3.25 (1.94, 5.43) | **<0.0001** |  |  |
| Negative Symptoms | 0.46 (0.22,0.97) | **0.041** |  |  |
| Aware of Condition | 0.41 (0.25,0.69) | **0.0008** |  |  |
| Adherence to medication | 0.23 (0.134,0.39) | **<0.0001** |  |  |
| Age | 0.97 (0.95,0.99) | **0.020** |  |  |

**Discussion**

The identification of alcohol use, tobacco smoking, and male sex as factors independently associated with substance use in schizophrenia underscores critical intersections between behavioural, biological, and social factors in this population. These findings align with existing literature that positions substance use as both a coping mechanism and a contributor to the complex psychopathology of schizophrenia. The substance use prevalence of 31.1% in our cohort is lower than SSA averages (58–65%) [14] but aligns with Nigerian (43%) [15] and Ethiopian (46.8%) [16] studies. This discrepancy may reflect Zambia’s strict substance criminalization or underdetection in records. Crucially, alcohol and tobacco smoking were dominant correlates, mirroring global patterns where 60–80% of persons with schizophrenia use tobacco [17] and 30–50% misuse alcohol [18]. Neurobiological mechanisms such as nicotine’s transient amelioration of cognitive deficits [19,20] and socioeconomic drivers like kachasu affordability likely underpin this. Alcohol and tobacco may serve dual roles: temporarily alleviating symptoms such as anxiety or cognitive deficits while perpetuating cycles of addiction through neurobiological pathways [21,22]. The prominence of these substances highlights the need to address polysubstance use as a multifaceted challenge rather than isolated behaviors.

*Factors Associated with Lower Odds of Substance Use in Multivariable Analysis*

The identification of female sex, marriage, higher education, and parental loss as factors associated with lower odds of substance use in adjusted models reveals nuanced socio-familial buffers against substance use. The robust association of female sex (AOR=0.27) aligns with global epidemiological patterns [23,24] but contrasts with Jordanian data where male gender dominated risk profiles [25]. This underscores gender-specific social norms in Zambia, where cultural stigmatization may disproportionately deter substance use among women. Marriage retained significance after adjustment (AOR=0.39), suggesting spousal support may mitigate isolation-driven substance use, though this effect is attenuated when substance-related behaviours are entrenched. Unexpectedly, having both parents deceased was associated with lower odds of substance use (AOR=0.29), potentially reflecting early resilience-building or alternative kinship support, a finding warranting qualitative exploration [10,26].

The persistence of male sex as a factor associated with substance use raises important questions about gender-specific vulnerabilities. A wide confidence interval for sex variable reflects limited female substance users (n=13), reducing precision. Despite this, the strong effect size (AOR>5) and statistical significance (p=0.003) support male gender as a key correlate. Biological differences, such as hormonal influences on dopamine regulation, may be linked to higher addiction rates. Sociocultural factors, including gendered norms around substance use and disparities in healthcare-seeking behaviour, likely further amplify this association [9,27]. This duality suggests that interventions must extend beyond clinical settings to address broader societal influences, particularly in communities where male substance use is normalized.

*Novel correlate: Hospitalization Frequency*

The emergence of hospitalization frequency as a variable associated with substance use (AOR=1.29 per admission) signals a clinically critical pattern. This aligns with several studies linking repeated hospitalizations to severe dual-diagnosis trajectories [24,28], suggesting that each admission may represent accumulated neurobiological vulnerability or systemic failures in continuity of care. In LUTH’s resource-constrained setting, this association may also reflect cyclical crises where substance use precipitates relapse, driving rehospitalization, a feedback loop demanding integrated inpatient substance interventions.

*Dissociation of Bivariate Predictors*

The loss of significance for violent behavior, negative symptoms, awareness of illness, and medication adherence in adjusted models highlights confounding pathways. For instance, violence (OR=3.25, p<0.0001 bivariately) may stem from substance-induced impulsivity rather than schizophrenia psychopathology, consistent with neurobiological models of addiction-psychosis interactions [29,30]. Similarly, medication non-adherence likely operates downstream of substance use (e.g., cognitive impairment from alcohol), explaining its attenuation when substance behaviours are modelled. The non-retention of negative symptoms contradicts the "self-medication" hypothesis [30], implying transient symptom alleviation by substances does not translate to population-level protection.

*Clinical and Policy Implications*

The association of education (AOR=0.44) advocates for socioeconomic interventions like vocational training to disrupt poverty-substance use cycles. Similarly, marital stability and gender-specific associations necessitate family-inclusive counselling and male-targeted harm reduction. The hospitalization finding demands systemic reforms: embedding addiction specialists in inpatient psychiatry teams and post-discharge monitoring. Limitations include recall bias in hospitalization documentation and ASSIST’s potential cultural insensitivity to local substances like *kachasu*.

Clinically, these findings advocate for integrated care models that concurrently address schizophrenia and substance use disorders. Programs emphasizing harm reduction, such as smoking cessation paired with psychosocial support, could disrupt the cycle of addiction while improving mental health outcomes. Gender-sensitive approaches, particularly for males, should be prioritized in resource allocation and policy design. Additionally, the protective roles of marriage and education underscore the importance of holistic assessments that consider socioeconomic, familial, and cultural contexts in treatment planning. This study has important limitations that warrant careful consideration. First, the cross-sectional design inherently precludes causal inference; while we identified significant associations (e.g., between alcohol use and substance use), we cannot determine whether substance use exacerbates schizophrenia symptoms or emerges as a consequence of untreated psychosis. Second, recall and documentation biases may have led to underreporting of substance use. In Zambia’s highly stigmatized cultural setting, where substance use carries legal repercussions and mental illness is often attributed to spiritual causes, patients may conceal illicit drug use. Clinician time constraints at LUTH, a high-volume referral hospital, further limit thorough documentation, particularly for nuanced behaviors like traditional substance consumption (e.g., *kachasu*). Third, although the ASSIST tool is WHO-validated globally, its performance in Zambia remains unstudied. Local substances (e.g., traditionally brewed alcohols, herbal mixtures) may not align with ASSIST’s standardized thresholds, potentially misclassifying culturally specific usage patterns. Fourth, our recruitment from LUTH, a tertiary urban center, introduces selection bias. Findings may not generalize to rural communities, where most Zambians reside and face greater barriers to mental healthcare, including limited psychiatric services and stronger reliance on traditional healers. Finally, unmeasured confounders such as socioeconomic status (e.g., household income), childhood trauma, or detailed employment history could influence both substance use and schizophrenia severity but were not captured in medical records, leaving residual confounding in our models.

**Conclusion**

This study found a prevalence of substance use of 31.1% among persons with schizophrenia at LUTH. Male gender, alcohol use, and tobacco smoking were associated with substance use, but multivariable analysis reveals associations for female sex, marriage, parental loss, and education with lower odds of use, alongside hospitalization frequency as a novel correlate. To mitigate this comorbidity, we recommend: (1) integrating gender-specific substance screening in psychiatric care, (2) socioeconomic programs targeting education and employment, (3) family-centered interventions leveraging marital and kinship support, and (4) specialized addiction services for frequently hospitalized patients. Validating assessment tools for Zambian cultural contexts and longitudinal studies to clarify causality are essential next steps.

**Availability of data and materials**

The raw data underlying the results presented in the study have been uploaded as supporting information 2.

**Competing interests**

The authors declare no competing interest exist.

**Funding**

This study received no funding

**References**

1.	Incidence, prevalence, and global burden of schizophrenia - data, with critical appraisal, from the Global Burden of Disease (GBD) 2019 - PubMed. [cited 1 May 2025]. Available: https://pubmed.ncbi.nlm.nih.gov/37500825/

2.	R T, W G, Dm B, J B, Re G, S H, et al. Definition and description of schizophrenia in the DSM-5. Schizophrenia research. 2013;150. doi:10.1016/j.schres.2013.05.028

3.	Prevalence estimates of substance abuse in schizophrenia and their correlates - PubMed. [cited 1 May 2025]. Available: https://pubmed.ncbi.nlm.nih.gov/1522409/

4.	(PDF) Prevalence of Substance Abuse in Patients with Schizophrenia. ResearchGate. [cited 1 May 2025]. doi:10.7860/JCDR/2020/43287.13577

5.	Alexander L. Mental Health in Zambia: 5 Facts. In: The Borgen Project [Internet]. 1 Sept 2023 [cited 7 July 2025]. Available: https://borgenproject.org/mental-health-in-zambia-2/

6.	Mayeya J, Chazulwa R, Mayeya PN, Mbewe E, Magolo LM, Kasisi F, et al. Zambia mental health country profile. Int Rev Psychiatry. 2004;16: 63–72. doi:10.1080/09540260310001635113

7.	Addiction and schizophrenia - PubMed. [cited 1 May 2025]. Available: https://pubmed.ncbi.nlm.nih.gov/10881208/

8.	Alcohol dependence and schizophrenia: what are the interrelationships? - PubMed. [cited 1 May 2025]. Available: https://pubmed.ncbi.nlm.nih.gov/8974370/

9.	Consortium SWG of the PG, Ripke S, Neale BM, Corvin A, Walters JT, Farh K-H, et al. Biological Insights From 108 Schizophrenia-Associated Genetic Loci. Nature. 2014;511: 421. doi:10.1038/nature13595

10.	J V, Am G, J D-R, M L, Pm L, F S, et al. [Environmental risk factors for schizophrenia: a review]. L’Encephale. 2013;39. doi:10.1016/j.encep.2011.12.007

11.	Substance abuse in schizophrenia. A Singapore perspective - PubMed. [cited 1 May 2025]. Available: https://pubmed.ncbi.nlm.nih.gov/12111024/

12.	Substance abuse treatment for people with schizophrenia - PubMed. [cited 1 May 2025]. Available: https://pubmed.ncbi.nlm.nih.gov/9801714/

13.	World Mental Health Report 2022 – Centre for Knowledge and Development. [cited 1 May 2025]. Available: https://www.ipeckd.org/world-mental-health-report-2022/

14.	Onaolapo OJ, Olofinnade AT, Ojo FO, Adeleye O, Falade J, Onaolapo AY. Substance use and substance use disorders in Africa: An epidemiological approach to the review of existing literature. World J Psychiatry. 2022;12: 1268–1286. doi:10.5498/wjp.v12.i10.1268

15.	Emmanuel GO, Akinsolu FT, Abodunrin OR, Ezechi OC. Prevalence and patterns of substance use in West Africa: A systematic review and meta-analysis. PLOS Glob Public Health. 2024;4: e0004019. doi:10.1371/journal.pgph.0004019

16.	Kibru B, Tesfaw G, Demilew D, Salelew E. The Prevalence and Correlates of Social Anxiety Symptoms among People with Schizophrenia in Ethiopia: An Institution-Based Cross-Sectional Study. Schizophr Res Treatment. 2020;2020: 3934680. doi:10.1155/2020/3934680

17.	Nesvåg R, Knudsen GP, Bakken IJ, Høye A, Ystrom E, Surén P, et al. Substance use disorders in schizophrenia, bipolar disorder, and depressive illness: a registry-based study. Soc Psychiatry Psychiatr Epidemiol. 2015;50: 1267–1276. doi:10.1007/s00127-015-1025-2

18.	Yapici Eser H, Kacar AS, Kilciksiz CM, Yalçinay-Inan M, Ongur D. Prevalence and Associated Features of Anxiety Disorder Comorbidity in Bipolar Disorder: A Meta-Analysis and Meta-Regression Study. Front Psychiatry. 2018;9: 229. doi:10.3389/fpsyt.2018.00229

19.	Chinyama J, Menon AJ. Mental Health and Healthcare Provision in Zambian Correctional Facilities. Medical Journal of Zambia. 2020;47: 208–214. doi:10.55320/mjz.47.3.88

20.	Zyambo C, Phiri MM, Zulu R, Mukupa M, Mabanti K, Matenga TFL, et al. Illicit alcohol consumption and its associated factors among patrons in Zambia: a cross-sectional analytical study. Front Public Health. 2025;13: 1444304. doi:10.3389/fpubh.2025.1444304

21.	Os J van, Rutten BP, Poulton R. Gene-Environment Interactions in Schizophrenia: Review of Epidemiological Findings and Future Directions. Schizophrenia Bulletin. 2008;34: 1066. doi:10.1093/schbul/sbn117

22.	Sa S, Rm M. Non-Genetic Factors in Schizophrenia. Current psychiatry reports. 2019;21. doi:10.1007/s11920-019-1091-3

23.	Chang S, Jeyagurunathan A, Lau JH, Shafie S, Samari E, Cetty L, et al. Problematic Drug Use Among Outpatients With Schizophrenia and Related Psychoses. Front Psychiatry. 2021;12. doi:10.3389/fpsyt.2021.762988

24.	Tumenta T, Oladeji O, Gill M, Khan BA, Olayinka O, Ojimba C, et al. Substance Use Patterns and Schizophrenia Spectrum Disorders: A Retrospective Study of Inpatients at a Community Teaching Hospital. J Clin Med Res. 2020;12: 803–808. doi:10.14740/jocmr4380

25.	Abbasi LN, Daradkeh TK, ElWasify M, Abassy S. Prevalence of substance use among a sample of patients attending an outpatient psychiatric clinic in Amman, Jordan. Discov Ment Health. 2024;4: 57. doi:10.1007/s44192-024-00113-1

26.	Schizophrenia Working Group of the Psychiatric Genomics Consortium. Biological insights from 108 schizophrenia-associated genetic loci. Nature. 2014;511: 421–427. doi:10.1038/nature13595

27.	Clinical aspects of substance abuse in persons with schizophrenia - PubMed. [cited 1 May 2025]. Available: https://pubmed.ncbi.nlm.nih.gov/12635559/

28.	Bennett ME, Brown CH, Peer J, Li L, Bellack AS. Patterns and Predictors of Changes in Substance Use in Individuals with Schizophrenia and Affective Disorders. J Dual Diagn. 2012;8: 2–12. doi:10.1080/15504263.2012.647466

29.	Ward HB, Nemeroff CB, Carpenter L, Grzenda A, McDonald WM, Rodriguez CI, et al. Substance use disorders in schizophrenia: Prevalence, etiology, biomarkers, and treatment. Personalized Medicine in Psychiatry. 2023;39–40: 100106. doi:10.1016/j.pmip.2023.100106

30.	Khokhar JY, Dwiel L, Henricks A, Doucette WT, Green AI. The Link Between Schizophrenia and Substance Use Disorder: A Unifying Hypothesis. Schizophr Res. 2018;194: 78–85. doi:10.1016/j.schres.2017.04.016

S1. Strobe checklist

S2. Data

S1 Table 1
S1. Table 2

S1. Table 3
