# Supplementary material for: Prevalence and factors associated with illicit substance use among persons with Schizophrenia at a Tertiary Referral Hospital in Zambia
Source: PLOS Glob Public Health. 2026 Jan 12;6(1):e0005024. doi: 10.1371/journal.pgph.0005024 (PMC12795356; doi:10.1371/journal.pgph.0005024)
Supplement: Attachment — Submitted filename: Manuscript Review Report.docx [file pgph.0005024.s010.docx]

**Peer Review Report**

**Title:** Prevalence and Factors Associated with Substance Use Among Persons with Schizophrenia at a Tertiary Referral Hospital in Zambia

**Summary:**

This manuscript investigates the prevalence and associated factors of substance use among persons with schizophrenia at the University Teaching Hospital (LUTH) in Zambia. The study is timely and relevant, providing valuable insights into a largely under-studied context in Sub-Saharan Africa. The prevalence of 31.1% substance use among schizophrenia patients is a key finding, alongside associated sociodemographic and clinical factors. The study addresses a significant gap in the literature, but several areas need clarification, refinement, and restructuring before the manuscript can be considered for publication.

**Major Comments:**

**1. Title and Abstract**

The title is clear and representative of the content. However, ensure consistency in capitalization ("Substance Use" instead of "Substance use").

The abstract is comprehensive but overly detailed in methods and results. Consider streamlining for clarity and balance.

**2. Introduction**

The introduction provides a strong rationale but could be more concise. Several references are cited but require formatting according to journal guidelines.

There is some repetition regarding the Zambian context (e.g., shortage of psychiatrists, stigma). Condense for better flow.

**3. Methods**

There is inconsistency: The abstract mentions a prospective cross-sectional study (Jan–June 2023), but the main methods describe a retrospective review (2021–2024). This must be clarified.

Eligibility criteria are stated clearly, but some duplication is present (criteria repeated twice). Please streamline.

Ethical approval details are appropriate; however, consider moving power calculation details to supplementary material.

**4. Results**

Results are well-structured with tables and figures. However, Table 1 and Table 2 formatting should be standardized (consistent decimal places, alignment of percentages).

Figures (flow diagram, correlation matrix) are referenced but not included in this version of the manuscript. Ensure they are provided.

The logistic regression section is detailed but could be condensed for readability.

**5. Discussion**

The discussion is thorough but overly lengthy and repetitive in some sections. Summarize global comparisons more concisely.

The novel finding of hospitalization frequency is valuable and should be highlighted earlier in the discussion.

Limitations are appropriately acknowledged, but consider emphasizing the reliance on secondary data as a primary limitation.

**6. Conclusion**

-The conclusion is strong, but avoid restating too many results. Focus on implications and recommendations.

**Minor Comments:**

1. Consistency issues: "Substance use" vs. "Substance Use"; "Livingstone" vs. "Lusaka" (clarify LUTH’s location—it is in Lusaka, not Livingstone).

2. Some references are cited as "[cited May 2025]" which may not align with submission timelines. Adjust for accuracy.

3. Grammar and phrasing: e.g., "share co-first authorship" should be "shared co-first authorship."

4. Ensure tables and supplementary files follow journal formatting guidelines.

**Recommendation:**

Major Revision. The study is important, but clarification of methods, removal of redundancy, and careful editing for consistency are needed.

**Reviewer:**

Dr Fathi Ali Araye Psychiatrist
