# Supplementary material for: Prevalence and factors associated with illicit substance use among persons with Schizophrenia at a Tertiary Referral Hospital in Zambia
Source: PLOS Glob Public Health. 2026 Jan 12;6(1):e0005024. doi: 10.1371/journal.pgph.0005024 (PMC12795356; doi:10.1371/journal.pgph.0005024)
Supplement: Attachment — Submitted filename: Responses to reviewers.docx [file pgph.0005024.s011.docx]

Responses to reviewers

1. If the authors have adequately addressed your comments raised in a previous round of review and you feel that this manuscript is now acceptable for publication, you may indicate that here to bypass the “Comments to the Author” section, enter your conflict of interest statement in the “Confidential to Editor” section, and submit your "Accept" recommendation.

Reviewer #3: All comments have been addressed

Reviewer #4: All comments have been addressed

Reviewer #5: (No Response)

Reviewer #6: All comments have been addressed

Reviewer #7: (No Response)

**RESPONSE: Thank you.**

**Reviewer #3:**

Abstract
Line 29 -31: Objective should be revised as “This study determined the prevalence and factors associated with substance use among adults with schizophrenia at the University Teaching Hospital (LUTH) in Zambia

**RESPONSE:** We thank the reviewer for this suggestion. We have revised the objective statement in the Abstract as recommended.

Introduction
Line 99 -101: The suggested study objective is “The study determined the prevalence and factors associated with substance use among adults with schizophrenia at the University Teaching Hospital (LUTH) in Zambia”.

**RESPONSE:** We have revised the objective statement in the Introduction as recommended.

Results
Line 304: The statement “Conversely, Alcohol Use was strongly associated with substance use”, what does this mean? Alcohol is also a substance!!!

**RESPONSE:** We thank the reviewer for this astute observation. To avoid confusion, we have revised the terminology throughout the manuscript. The primary outcome is now explicitly defined as "illicit substance use" (referring to cannabis, cocaine, opioids, etc.), to distinguish it from the separate exposure variables of alcohol and tobacco use. The sentence in question has been rephrased for clarity.

**Reviewer #4:**

Major Revision. The study is important, but clarification of methods, removal of redundancy, and careful editing for consistency are needed.

**RESPONSE:** We thank the reviewer for recognizing the importance of our study. We have undertaken a thorough revision of the manuscript to enhance clarity, remove redundancy, and ensure consistency in terminology and reporting, as detailed in our responses to the specific points raised by all reviewers.

**Reviewer #5:**

Summary and Overall Assessment
This manuscript is well-crafted and presents meaningful findings. The study is methodologically robust, and the results are clearly articulated. It makes a valuable contribution to the field, enhancing our understanding of the topic. I recommend minor revisions to enhance clarity, formatting, and alignment with the journal's guidelines before final acceptance.
Strengths
• The research objectives are clearly stated, and the methodology is appropriately designed.
• The results are logically organised and supported by relevant data.
• The discussion thoughtfully integrates the findings with existing literature and highlights important public health implications.

**RESPONSE: Thank you for the positive comment on our manuscript. Thank you for all comemnts that have helped us improve it**

Areas for Improvement
- Abstract Length: The abstract currently exceeds the 300-word limit set by PLOS Public Health. Please revise it to be more concise while retaining essential information.

**RESPONSE: We have carefully revised the abstract to be more concise, reducing it to meet the 300-word limit of PLOS Public Health while retaining all essential information.**

- Reference Formatting: Some references are missing DOI numbers. Kindly verify and update all references to include DOIs where available.

**RESPONSE: We have verified and updated all references to include DOI numbers where available.**

- Language and Style: Minor grammatical and typographical errors are present throughout the manuscript. A thorough proofreading is recommended.

**RESPONSE: We have performed a thorough proofreading of the entire manuscript to correct minor grammatical and typographical errors.**

Final Remarks
Overall, this is a well-structured and informative study that offers valuable insights for public health research. With the above minor revisions, the manuscript will be well-positioned for publication in PLOS Public Health.

**RESPONSE: Thank you**

**Reviewer #6:**

Congratulations to the authors! This is a very well-written paper, and all previous comments have been addressed appropriately.

Response: thank you

I have very minor comments for the authors:
1) Clarify that you are assessing psychoactive substance use earlier in the paper, as substance use can sometimes be an umbrella term and include alcohol and/or tobacco use. This can be a bit confusing and the first mention of psychoactive substance use is in the methods.

**RESPONSE: We thank the reviewer for this excellent suggestion. We have now clarified the specific focus on "illicit substance use" in the Abstract, Introduction, and Methods sections to avoid any ambiguity with the broader term "substance use."**

2) Cite the software used for analysis.
**RESPONSE: We have now explicitly cited the software used for analysis (StatCrunch) in the Data Analysis section.**

3) Cite the statistical standards and threshold described in the methods.

**RESPONSE: We have added a sentence in the Data Analysis section to explicitly cite the statistical standards and thresholds used for significance and model fitting.**

Other than these, I think this paper is ready.

**RESPONSE: thank you**

**Reviewer #7:**

This cross-sectional study explores substance use among persons with schizophrenia in Zambia, addressing a critical evidence gap in both the local and broader sub-Saharan African literature. The study offers valuable insights into a population at high risk for substance use disorders.

Main Strengths

1. The study addresses a clear evidence gap within Zambia’s mental health system.

2. It meets STROBE reporting standards, enhancing transparency and reproducibility.

3. Clinical and sociodemographic variables were appropriately selected and comprehensively assessed.

4. Statistical methods were rigorous, with multivariable modeling, collinearity diagnostics, and model fit procedures clearly reported.

**RESPONSE: Thank you for your positive and constructive feedback which has improved our manuscript**

Points for Improvement

1. In broader literature, “substance use” typically includes tobacco, alcohol, and other drugs. Excluding tobacco and alcohol may confuse readers. A more accurate term might be illicit substance use to reflect the study's classification of other drugs.

**RESPONSE:** We agree with the reviewer that the term "substance use" can be ambiguous. As noted in our response to Reviewer #3, we have changed the primary outcome terminology to "illicit substance use" throughout the manuscript to accurately reflect the study's focus on substances other than alcohol and tobacco.

2. The final logistic regression model demonstrated poor calibration (Hosmer-Lemeshow p = 0.0196), raising concerns about the model’s fit. The reliability of the associations could be improved by re-estimating the model using penalized regression or by reducing the number of predictors.

**RESPONSE:** We acknowledge the reviewer's concern regarding the model's calibration (H-L p=0.0196). We have added a statement in the Results and Discussion acknowledging this limitation. We have tried to implement additional analyses , however, we have retained the original model for its strong discriminative ability (83% accuracy) and clinical relevance justify its presentation, with the calibration issue noted as an area for future research. We thank the reviewer for highlighting this as a limitation

3. Continuous predictors like age and number of hospitalizations were modeled linearly. However, their relationship with substance use may be non-linear. The analysis would benefit from assessing this assumption using methods such as the Box-Tidwell test or by modeling non-linearity with splines or categorized versions.

**RESPONSE:** We thank the reviewer for this insightful suggestion. We have acknowledged this as a limitation in the Discussion, stating that the assumption of linearity for continuous variables was not tested and that future studies could benefit from exploring non-linear relationships.

4. While adjusted predictors are clearly reported, some variables significant in bivariate analysis (e.g., negative symptoms, awareness of diagnosis) were omitted from the final model without explanation. Clarifying whether these were excluded based on stepwise selection or clinical irrelevance would enhance transparency.

**RESPONSE:** We have enhanced the transparency of our variable selection process by adding a more detailed explanation in the Data Analysis section, clarifying that the final model was derived through backward elimination based on statistical significance (p<0.05) and model fit indices (AIC), which led to the exclusion of some bivariately significant variables.

Minor Comments

Descriptive tables should include 95% confidence intervals for proportions to improve interpretability and comparability across groups.

**RESPONSE:** We have added 95% Confidence Intervals for the proportions in Table 1 as requested, improving the interpretability of the descriptive data.
