# Supplementary material for: Prevalence and factors associated with illicit substance use among persons with Schizophrenia at a Tertiary Referral Hospital in Zambia
Source: PLOS Glob Public Health. 2026 Jan 12;6(1):e0005024. doi: 10.1371/journal.pgph.0005024 (PMC12795356; doi:10.1371/journal.pgph.0005024)
Supplement: Attachment — Submitted filename: recommendation letter.docx [file pgph.0005024.s012.docx]

**Date:** 17^th^ / Dec / 2025

**To:**
The Editorial Team
*PLOS Global Public Health*

**Subject:** Recommendation and Endorsement of Revised Manuscript for Publication

Dear Editors,

I am writing to formally recommend the revised manuscript entitled “Prevalence and Factors Associated with Illicit Substance Use Among Persons with Schizophrenia at a Tertiary Referral Hospital in Zambia” for consideration for publication in *PLOS Global Public Health*.

I have carefully reviewed the revised version of this manuscript and the authors’ responses to the reviewers’ and editorial comments. In my assessment, the authors have satisfactorily addressed all comments and concerns raised during the review process. The revisions have significantly strengthened the manuscript in terms of methodological clarity, analytical rigor, presentation of results, and interpretation of findings. All major and minor issues previously identified have been adequately resolved.

The study is scientifically sound, ethically conducted, and methodologically robust. It addresses an important and underexplored public health problem in a low-resource setting by providing novel data on the prevalence and correlates of illicit substance use among persons with schizophrenia in Zambia. To my knowledge, this represents the first comprehensive study on this topic in the Zambian context, filling a critical gap in regional and global mental health literature.

The use of validated tools, appropriate statistical analyses, and clear reporting in line with STROBE guidelines enhances the credibility of the findings. Ethical approval was appropriately obtained, and data confidentiality and integrity were maintained throughout. The discussion is balanced, evidence-based, and places the findings within a relevant global and regional public health context, with practical implications for integrated mental health and substance-use interventions.

In my professional opinion as a psychiatrist, and based on my review of the revised manuscript, this study now meets the scientific, ethical, and reporting standards required for publication in *PLOS Global Public Health*. I therefore consider the manuscript acceptable for publication, subject only to the journal’s final editorial decision.

Thank you for considering this recommendation.

Yours sincerely,

**Dr. Fathi Ali Araye**
Consultant Psychiatrist
Email: faatixyare50@gmail.com
Signature: fathi
