# Supplementary material for: Prevalence and factors associated with illicit substance use among persons with Schizophrenia at a Tertiary Referral Hospital in Zambia
Source: PLOS Glob Public Health. 2026 Jan 12;6(1):e0005024. doi: 10.1371/journal.pgph.0005024 (PMC12795356; doi:10.1371/journal.pgph.0005024)
Supplement: Attachment — Submitted filename: RESPONSES TO EDITOR AND REVIEWER COMMENTS.docx [file pgph.0005024.s008.docx]

**RESPONSES TO EDITOR AND REVIEWER COMMENTS**

**Editor Comment 1:** The manuscript contains causal language (e.g., “predictors”), which is inappropriate given the cross-sectional design. Such statements are misleading and should be removed. The authors should revise these statements to reflect correlational findings.
**Response:** We thank the editor for this critical feedback. We have carefully reviewed the entire manuscript and **replaced all causal language (e.g., "predictors," "risk factors," "protective factors," "drivers," "increased risk") with correlational and associative language (e.g., "factors associated with," "correlates," "linked to," "were associated with")**. These changes are now reflected in the title, abstract, results, discussion, and conclusion.

**Editor Comment 2:** The use of labeling and stigmatizing language is not accepted. Stigmatizing phrases, such as “schizophrenic patients”, should all be removed throughout the paper. The title should also be revised accordingly.
**Response:** We sincerely apologize for this oversight. We have replaced all instances of "schizophrenic patients" with person-first language, specifically **"patients with schizophrenia"**. The title has been revised to **"Prevalence and Factors Associated with Substance Use Among Persons with Schizophrenia at a Tertiary Referral Hospital in Zambia"**.

**Editor Comment 3:** Clear objectives and hypotheses should be formulated at the end of the introduction. Inclusion criteria should be further detailed (outpatients or inpatients? clinical remission? schizophrenia according to which criteria? who made the diagnosis?).
**Response:** We have added a clear objectives and hypotheses section at the end of the introduction. We have also significantly expanded the 'Eligibility and Recruitment' subsection within the Methods to provide the requested details on inclusion criteria, diagnosis, and clinical status.

**Editor Comment 4:** The methods section is poorly informed. More details are required about how clinical variables were assessed (hallucinations, delusions, disorganized speech, disorganized behavior, negative symptoms, cognitive impairment, suicidality and self-harm).
**Response:** Thank you for this suggestion. We have added a new subsection titled **"Assessment of Clinical Variables"** under the main 'Methods' section to provide detailed descriptions of how each clinical variable was defined and ascertained from the medical records.

**Reviewer #3 Comment 1 (Abstract):** The objective in the abstract should be complete (see the title). The conclusion drawn by the authors in the abstract are not in line with the objective and the title. Therefore, the authors need to paraphrase the conclusion. The authors need to be clear on the sentence (line 35-36), “Data were collected from medical records including records of structured interviews with validated tools…”, were the structured interviews information retrieved also from records or from the medical records, that is when the study participants were contacted for the interview? The authors need to clarify on this.
**Response:** We have revised the abstract objective to mirror the title more completely. We have paraphrased the conclusion to ensure it aligns with the stated objective and title by focusing on the key associations found. We have also clarified the data collection method to explicitly state that **all data, including from previously conducted structured interviews, were retrieved from existing medical records** and that no new patient interviews were conducted for this study.

**Reviewer #3 Comment 2 (Introduction):** The authors need to improve the grammar on the sentence (Line 88-91). And the objective of the study also needs to be improved i.e. stated clearly for easy understanding.
**Response:** We have rephrased the indicated sentence for improved grammar and clarity. We have also rewritten the study objective at the end of the introduction to be more precise and clear.

**Reviewer #3 Comment 3 (Methods):** According to the study design, which is the retrospective cross-sectional study, as mentioned above “Data were collected from medical records including records of structured interviews with validated tools…”, were the structured interviews information retrieved also from records or from the medical records, that is when the study participants were contacted for the interview? The authors need to clarify on this. Line 144, no patient interviews were conducted; the authors need to clear how information from the structured interviews mentioned in the abstract were obtained. Line 121, the authors should avoid starting a sentence with a value.
**Response:** We have clarified in both the abstract and methods that the information from structured interviews (e.g., ASSIST, PANSS) was **documented in the medical records as part of routine clinical care** and was retrospectively extracted for this study. **No new interviews were conducted.** We have also rephrased the sentence that began with a value.

**Reviewer #3 Comment 4 (Results):** Line 296, the authors mention “smoking”, which smoking is being referred in the paper?
**Response:** We have specified throughout the manuscript that "smoking" refers to **tobacco smoking**. This has been clarified in the abstract, methods, results, and discussion.

**Reviewer #3 Comment 5 (Conclusion):** The authors should also conclude on the prevalence since it is key in the title.
**Response:** We have revised the conclusion to explicitly mention the prevalence found in our study.
